# Ultra-Wide-Field OCT Measurements in Patients with Age-Related Macular Degeneration in Relation to Their Visual Function

**DOI:** 10.3390/diagnostics14242868

**Published:** 2024-12-20

**Authors:** Maciej Gawęcki, Krzysztof Kiciński, Jan Kucharczuk, Sławomir Teper, Magdalena Hubert, Tomasz Kuc

**Affiliations:** 1 Department of Ophthalmology, Pomeranian Hospitals, 84-200 Wejherowo, Poland; krzysztofkg999@icloud.com (K.K.); magdalenahubert111@gmail.com (M.H.); tomasz_kuc@wp.pl (T.K.); 2 Dobry Wzrok Ophthalmological Center, 80-392 Gdansk, Poland; 3 Department of Ophthalmology, 10th Military Research Hospital and Polyclinic, 85-681 Bydgoszcz, Poland; jankucharczuk@wp.pl; 4 Chair and Clinical Department of Ophthalmology, Faculty of Medical Sciences in Zabrze, Medical University of Silesia, 40-752 Katowice, Poland

**Keywords:** ultra-wide-field optical coherence tomography, age-related macular degeneration, retinal thickness, choroidal thickness

## Abstract

Background: Ultra-wide-field optical coherence tomography (UWF-OCT) devices have recently been introduced to clinical practice. The goal of this study was to compare choroidal and retinal thickness (CT and RT) in age-related macular degeneration (AMD) with a healthy control group using UWF-OCT Xephilio S1. Additionally, we sought to determine the relationship between the RT and CT of patients with AMD, measured in different sectors, and their visual acuity. Methods: The study included 104 eyes from 74 participants with dry AMD, 119 eyes from 86 participants with wet AMD, and 85 eyes from 53 healthy controls. Of the participants with wet AMD, 87 eyes received anti-VEGF treatment, 13 were treatment naïve, and 19 had incomplete data. The analyzed measurements were taken in the central area of 3 mm in diameter and two peripheral rings located between 3–9 mm and 9–18 mm diameters. Results. There was no significant variation in the RT in any sector between the three study groups. CT in dry and wet AMD cohorts was significantly lower compared to controls in every sector. Patients with treatment-naïve wet AMD did not demonstrate significant CT loss but had a tendency for lower CT values. Visual impairment in patients with AMD correlated with older age in both subgroups and with smaller RT in the dry AMD subgroup. Conclusions: Values of RT and CT obtained at the mid- and far-periphery with UWF-OCT generally reflect the alterations observed in AMD in the central part of the posterior pole. Intravitreal anti-VEGF treatment might contribute to loss of choroidal tissue observed in AMD in every sector.

## 1. Introduction

The relationship between the thickness of the retina (RT) and the choroid (CT) and age-related macular degeneration (AMD) has been assessed in numerous studies involving standard field optical coherence tomography (OCT) devices [1,2,3]. Typically, standard spectral domain OCT (SD-OCT) provides measurements in scanning fields corresponding to early treatment diabetic retinopathy study (ETDRS) grid, that is, in concentric circles of 1, 3, and 6 mm in diameter. Thus, evaluation of the RT and CT is limited to the central portion of the posterior pole and, as such, does not provide a complete view of the retina and the choroid as broad anatomical structures. Modern ultra-wide-field OCT (UWF-OCT) enables measurement of areas of more than 20 mm in width [4]. Hence, it has the potential to provide information on the involvement of the peripheral retina and choroid in ophthalmic diseases predominantly affecting the macula, including AMD [5]. Evaluation of the retinal periphery in patients with AMD seems important in the light of previous research that confirmed the presence of peripheral pathology in this predominantly macular disorder. The authors, who used ultra-wide-field imaging in patients with AMD, such as UWF-fundus autofluorescence or UWF-color photography, reported the occurrence of peripheral drusen, retinal atrophy, or other AMD-like lesions in the majority of patients [6,7,8]. Observed lesions were more prevalent in the AMD cohort compared to the healthy group. These findings create a background for our analysis with the use of a new UWF modality.

The goal of the study was to compare CT and RT in patients with AMD measured with UWF-OCT in reference to a healthy control group. Additionally, we sought to determine the relationship between the RT and CT of patients with AMD, measured in different sectors, and their visual function (best-corrected visual acuity—BCVA).

## 2. Material and Methods

The study was performed in accordance with the tenets of the Declaration of Helsinki and approved by the ethical board of the Wzrok Ophthalmological Clinic (No. 5/2023). All procedures were conducted in the Department of Ophthalmology of Specialist Hospital in Chojnice between April and December 2023. The subjects included 74 participants with dry AMD (104 eyes), 86 participants with wet AMD (119 eyes), and 53 healthy controls (85 eyes). Data regarding intravitreal treatment were available for 100 eyes with wet AMD.

Patients with AMD, selected consecutively, were individuals treated or followed in the Polish National AMD Treatment Program. According to the rules of that system, all patients with an exudative form of AMD are treated with intravitreal anti-VEGF injections administered according to the protocol provided with the specific medication. The diagnosis of AMD was made on the basis of tests necessary for eligibility for the program. These included best-corrected visual acuity (BCVA), anterior and posterior segment slit lamp evaluation, intraocular pressure measurement, fluorescein angiography, and spectral domain optical coherence tomography (SD-OCT). OCT angiography was used in selected cases to help determine the exudative form of AMD. Cases of polypoidal choroidal vasculopathy (PCV) were excluded based on the SD-OCT and angio-OCT examinations [9].

Patients with significant opacity of ophthalmic media that precluded the acquisition of UWF-OCT scans were excluded from the study, as well as any with poor-quality scans (quality index below 6 on a 10-grade scale). Other exclusion criteria were present or past ophthalmic comorbidities that could influence the OCT measurements, such as epiretinal membranes, diabetic retinopathy, retinal vascular occlusions, and previous vitreoretinal surgery. 

The healthy control group included patients aged 50 years or older examined in the hospital’s outpatient clinic during routine screening visits. The control group was slightly younger compared to AMD groups; thus, all the performed analyses were adjusted for age. Only individuals with no ocular disease were included in the group; patients with opacities of ophthalmic media and amblyopic eyes were excluded.

**Figure 1:**
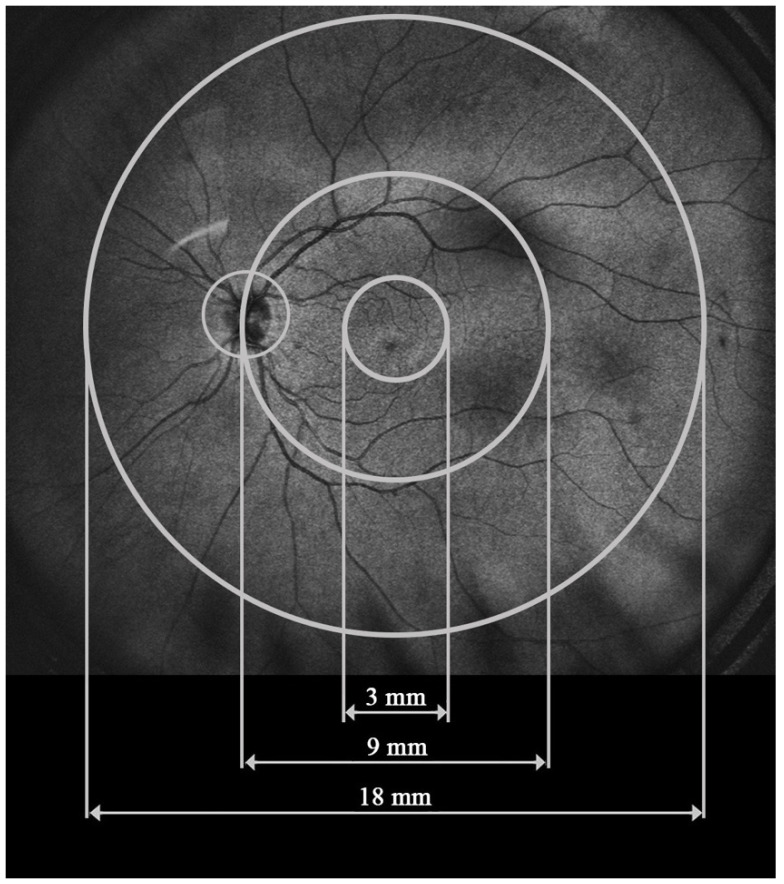
Retinal and choroidal zones analyzed in the study with UWF-OCT.

UWF-OCT testing was performed in all study participants after pupil dilation using a new swept-source device, the Xephilio OCT-S1 (Canon Medical Systems Europe B.V., 2023, Amstelveen, The Netherlands). This device provides scanning of the retina and choroid over a large area of 20 × 23 mm with a single acquisition and the speed of 10,000 A scans per second. The protocol for RT and CT measurements includes 25 fields enclosed in a large circular area of 18 mm in diameter. For the purpose of our study, the measured sectors were merged into three zones extending from the center to the periphery: the central circle, 3 mm in diameter (central), the ring between the central 9 mm circle and the central 3 mm circle (perifoveal), and the second, more peripheral ring between the central 18 mm and 9 mm circles (peripheral) (Figure 1). RT and CT values presented for each zone in our study stand for the arithmetic mean calculated from the values provided for smaller sectors by the Xephilio OCT-S1 device.

Retinal thickness was measured between the retinal pigment epithelium (RPE) and internal limiting membrane (ILM). Choroidal thickness was represented by the value of measurement between the Bruch’s membrane and the choroidal scleral interface. Each thickness value represents average retinal or choroidal thickness for a specific sector. The Xephilio OCT-S1 system uses approximation for the calculation of the thickness of curved surfaces. Moreover, the internal software provides eye axial length correction to change default pixel size according to the patient eye size. Corrections are also utilized for the peripheral areas, considering the eyeball to be a true sphere. Each scan was individually checked and corrected for the layer segmentation. Scans of poor quality were excluded from the analysis.

The study compared three study groups—dry AMD, wet AMD, and controls—according to RT and CT in the three sectors described above. For AMD cohorts, correlations between the BCVA and RT and CT in central and peripheral zones were evaluated. Analysis for the wet AMD subgroup also included correlation between the effects of intravitreal treatment on BCVA and RT and CT.

### Statistical Procedures

Categorical variables were shown as integer numbers and percentages. Numeric traits were evaluated by their mean, median, standard deviation, and lower-to-upper quartile values. The normality of distribution was assessed using the Shapiro–Wilk W test. Levene’s test was used to assess the homogeneity of variances. A multifactor analysis of variance (ANOVA) was performed to test the significance of differences in normally distributed numerical traits between the study groups. Generalized linear models (GLMs) were fitted when dealing with non-normally distributed numerical variables. All the multivariate procedures were controlled for the participants’ age and gender. Spearman’s rank correlation coefficients were computed when appraising relationships between selected numerical traits. A level of *p* < 0.05 was deemed statistically significant. All procedures were performed using Statistica™, release 13.3 (TIBCO Software Inc., Palo Alto, CA, USA).

## 3. Results

Table 1 presents baseline characteristics of the study groups.

No differences were noted between the study groups in gender distribution or axial length. Thus, possible effects of gender or ametropia on the OCT measurements, noted by other authors, were excluded [10]. The control group was slightly younger compared to AMD subgroups; thus, further statistical calculations were specifically adjusted for age and gender. Significant differences between the groups were noted for BCVA: both AMD subgroups had poorer BCVA compared to the healthy controls, and the wet AMD cohort had poorer final BCVA compared to the dry AMD subgroup (*p* < 0.0001 for each pair).

Table 2 shows characteristics of the study groups according to UWF-OCT measurements.

There were no significant variations between the analyzed sub-cohorts in reference to RT measured in all three zones. Distinct differences were observed in CT. The choroid was significantly thinner in every zone (central, perifoveal, and peripheral) in both patients with dry AMD and patients with wet AMD compared to healthy controls. No apparent differences in CT were noted between the AMD subgroups in any sector (Table 3).

**Figure 2:**
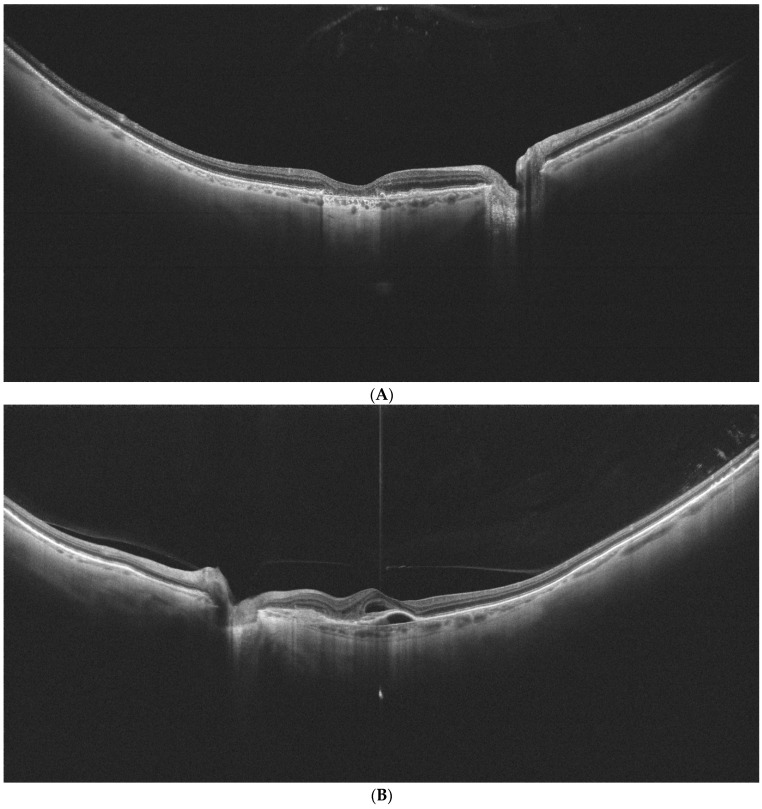

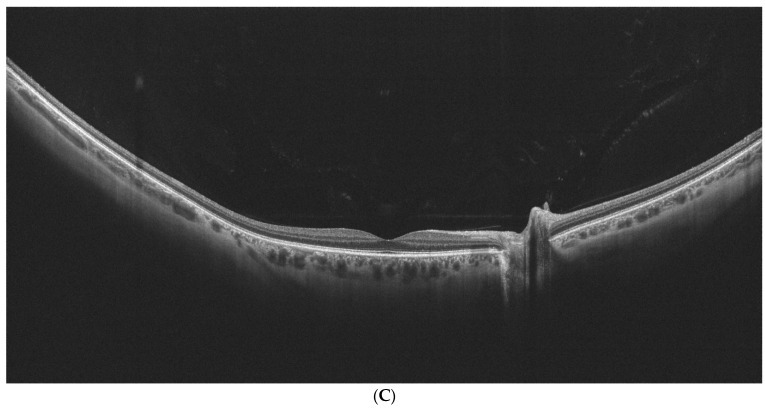
**(A**) is an example of UWF-OCT in geographic atrophy. Increased penetration of the retina is noted in the central part of the posterior pole. The choroid is distinctly thin. Retinal thickness values state 284 µm, 312 µm, and 227 µm respectively for the central, perifoveal, and peripheral zones. For the choroid, these thicknesses constitute 184 µm, 174 µm, and 152.75 µm, respectively. (**B**) Exudative form of AMD in UWF-OCT scan. Subretinal and sub-RPE fluid is observed in the retinal center. As in the previous scan, the choroid appears thin. Retinal thicknesses are observed at 345 µm in the central zone, 266.81 µm for the perifoveal, and 206 µm for the peripheral. Choroidal thickness values stand for 223 µm, 168.25 µm, and 139.25 µm, respectively, for the analyzed sectors. (**C**) UWF-OCT scan in a healthy individual. The choroid is apparently thicker compared to patients with AMD from (**A**,**B**). Retinal thickness measurements are noted at 351 µm, 299 µm, and 216.5 µm for central, perifoveal. and peripheral zones, respectively. Choroidal thickness values for these zones are, respectively, 398 µm, 328.87 µm, and 252.37 µm.

Figure 2A–C show examples of UWF-OCT images of AMD and healthy patients.

As application of anti-VEGF treatment may affect the choroid, separate analysis was performed for wet AMD sub-cohorts: treated versus treatment-naïve (Table 4 and Table 5). Patients who were treatment-naive were either cases just after diagnosis or not treated due to lack of consent or poor potential for improvement. Patients who were treated with anti-VEGF had significantly lower CT values compared to controls. Such significance was not noted for patients who were treatment-naïve, although their CT values are still low.

Table 6 presents correlations between RT and CT of patients with AMD and BCVA, duration of treatment, number of received intravitreal injections (IVI), and age. Significant correlation of the loss of visual acuity was noted only in the dry AMD group and only for the central zone of the retina (*p* = 0.0018). BCVA in dry AMD was not directly influenced by RT in other zones nor by variations of CT. No such correlation was found for the wet AMD group. BCVA significantly correlated with the age for patients with AMD for both wet and dry sub-types. Higher BCVA logMAR values (worse visual acuity) were strongly associated with older age (*p* = 0.009 for wet AMD and *p* = 0.001 for dry AMD).

Mean duration of anti-VEGF treatment for the wet AMD group was 26.05 ± 24.93 months. General estimation equations were used to evaluate changes in BCVA pre- versus post-treatment, including covariables such as age, gender, choroidal thicknesses, and duration of treatment. It was found that BCVA did not statistically significantly change after anti-VEGF treatment (*p* = 0.4949). There were no statistical relationships between the amount of BCVA change and the study participants’ demographics or choroidal thicknesses (for central CT: *p* = 0.6429, for perifoveal CT: *p* = 0.9436, and for peripheral CT: *p* = 0.9138). The number of received IVIs had no significant impact on RT and perifoveal and peripheral CT. However, a higher number of received IVIs correlated with a thicker central choroid. BCVA was negatively affected by the duration of therapy in the wet AMD cohort (*p* = 0.0421); however, as mentioned earlier, the change was not statistically significant. Significant loss of RT in wet AMD was also noted over time, but only in the perifoveal zone. This tendency was not confirmed for other retinal sectors or any of the choroidal sectors.

Strong and significant loss of RT and CT in patients with AMD is noted with older age. This refers to all retinal and choroidal zones of the dry AMD cohort and all but the central retinal sector for the wet AMD group. Visual impairment associated with both forms of AMD is strongly correlated with older age.

## 4. Discussion

Utilization of UWF-OCT for assessment of patients with AMD provides a broad view of the retinal and choroidal structures. Values of RT and CT obtained at the mid- and far-periphery generally reflect the alterations observed in the central part of the posterior pole and hence confirm the course of the processes associated with retinal degeneration. Our study results indicate that the choroid is generally significantly thinner in all sectors in patients with AMD compared to the healthy population, but RT remains similar in all three study groups. The type of AMD, wet or dry, does not affect CT significantly. Both dry and wet AMD cohorts present with similar CT values at all analyzed sectors.

Numerous studies employing standard field OCT have analyzed the relationship between CT and the development and progression of AMD, but with no consensus regarding such a relationship. A large study by Jonas et al. comparing patients with AMD with a healthy control group did not prove any relationship between CT in the foveal or perifoveal region and the presence of wet or dry AMD [11]. Manjunath et al. failed to prove any direct relationship between the duration or type of AMD and CT [12]. On the other hand, Ting et al. reported loss of CT and choroidal remodeling in patients with AMD with time in a 12-month observation [13]. This finding is consistent with our results.

Results regarding CT in wet AMD should be analyzed more closely in the context of received treatment, especially anti-VEGF. It has been reported that anti-VEGF therapy negatively influences CT [14]; however, this change does not depend on the number of received treatments or follow-up duration [15,16,17]. These findings suggest that decreased CT values might result from anti-VEGF treatment in addition to the degenerative process. In our study, patients treated with anti-VEGF had significantly lower CT values in all zones in comparison with healthy eyes. Despite that, a higher number of received IVI correlated with greater CT in the center. Such results are consistent with other reports, where patients with higher CT values at the beginning of therapy usually required more intravitreal injections during anti-VEGF treatment [18]. Treatment-naive individuals did not have a significantly lower CT compared to the healthy group. However, the treatment-naïve group was small, which could impact statistical outcomes. The difference of CT between patients who were treatment-naïve and patients who were treated did not reach statistical significance. It is plausible that application of anti-VEGF sustains an additional degenerative factor eliciting choroidal thinning, and CT loss in patients with treatment-naïve wet AMD cannot be excluded. Other authors have also proposed such hypotheses [19].

The relationship between the number of applications of anti-VEGF and CT is not straightforward, as shown in many studies [15,16,17]. Our research did not confirm a correlation between CT and treatment duration for any sector.

Other studies have noted a progression of subfoveal choroidal thinning with increased severity of non-exudative types of AMD in age-adjusted analysis [20]. Specifically, subfoveal CT is smaller in patients with early non-exudative AMD [21] as well as in individuals with geographic atrophy of retinal pigment epithelium (RPE) compared to controls [22]. These results are consistent with the conclusions of our study.

Our research shows a very strong correlation between CT loss in patients with AMD and increasing age. This finding was also reported earlier with the use of UWF-OCT for the healthy cohort [23]. It seems that in AMD, aging and degeneration overlap, adding to loss of choroidal tissue. Such a relationship is particularly strong for the retinal layer in patients with dry AMD. According to results of our study, aging and progression of AMD result in loss of retinal cells in every sector, including the far periphery. This process is well described in the literature based on standard field OCT [24]. The UWF-OCT enables the study of its extension and involvement of the peripheral areas. A deficit in the central retinal thickness has a direct correlation with the impairment of BCVA in the dry AMD cohort. This finding has also been reported in other studies [25,26].

Loss of retinal thickness with age is also observed in patients with wet AMD, but not for the central 3 mm circle, where we typically observe retinal edema due to neovascularization. That process, associated with central retinal thickness (CRT) increase, obscures the retinal tissue damage, and thus a correlation between CRT and BCVA is not observed.

Both dry and wet AMD subgroups show a strong correlation between worsening visual acuities and advanced age. This well-known finding confirms characteristics of AMD described in other large epidemiological studies [27,28,29,30].

To date, AMD has been analyzed in UWF-OCT in only one study related to different exudative forms of AMD in an Asian population, without reference to healthy individuals [31]. Thus, our results cannot be compared with other studies employing wide-field OCT equipment and must be evaluated by themselves.

## 5. Conclusions

Comparison of choroidal and retinal thicknesses in patients with dry AMD and patients with wet AMD versus healthy controls using UWF-OCT confirms loss of choroidal and retinal tissue at peripheral areas in AMD. Intravitreal anti-VEGF might contribute to loss of choroidal tissue in wet AMD, independently of the number of received treatments. Loss of central retinal thickness observed in dry AMD correlates strongly with visual impairment.

## Figures and Tables

**Table 1:** Baseline characteristics of the study cohort by AMD prevalence and type.

Analyzed Trait	Controls	Wet AMD	Dry AMD	*p* Value
No. of participants, *n* (%)	53 (24.88)	86 (40.38)	74 (34.74)	
No. of eyes, *n* (%)	85 (27.66)	119 (34.69)	104 (30.32)	
		87 (73.11) received IVI 13 (10.92) treatment-naïve 19 (15.97) incomplete data		
Gender, *n* (%):				
• Female	42 (58.33)	56 (65.12)	51 (68.92)	=0.0600
• Male	30 (41.67)	30 (34.88)	23 (31.08)	
Age (years)	68.13 ± 8.76	74.46 ± 6.96	74.35 ± 5.58	<0.0001
Axial length	24.65 ± 1.40	24.82 ± 1.67	24.93 ± 1.92	=0.4562
BCVA (logMAR) *	0.00 ± 0.00	B = 0.49 ± 0.21	0.21 ± 0.30	<0.0001
		F = 0.61 ± 0.44		

BCVA—best-corrected visual acuity; AMD—age-related macular degeneration; B—baseline; F—final. * Repeated measurements of BCVA in the wet AMD Group, *p* = 0.4949.

**Table 2:** Descriptive statistics for the retinal/choroidal thicknesses (µm) by AMD prevalence and type (*n* = 343 eyes), controlled for age and gender.

Foveal and Average Parafoveal Thicknesses [µm]	Study Group	Statistical Parameter				*p* Value
		M	SD	Me	Q_1_–Q_3_	
Retinal Thicknesses						
Foveal (Central)	Controls	334.69	21.65	334.00	325.00–348.00	=0.6945
	Wet AMD	329.56	53.57	318.00	293.00–355.00	
	Dry AMD	326.32	22.43	327.00	312.50–343.00	
Perifoveal	Controls	285.79	14.39	290.19	275.91–296.66	=0.1176
	Wet AMD	278.52	28.41	285.87	260.19–295.25	
	Dry AMD	284.55	15.61	282.50	275.22–295.06	
Peripheral	Controls	219.97	11.87	221.25	209.94–228.44	=0.1253
	Wet AMD	220.41	14.63	221.75	209.62–230.12	
	Dry AMD	221.72	12.90	221.72	212.25–230.25	
Choroidal Thicknesses						
Foveal (Central)	Controls	273.64	84.02	273.50	198.50–338.50	=0.0245
	Wet AMD	237.13	81.19	224.00	178.00–278.00	
	Dry AMD	244.01	89.89	233.50	181.00–285.50	
Perifoveal	Controls	235.97	69.30	237.50	177.12–284.06	=0.0080
	Wet AMD	195.74	56.02	186.25	152.81–225.62	
	Dry AMD	210.38	70.15	200.53	164.34–246.28	
Peripheral	Controls	177.51	45.96	166.62	143.31–203.37	=0.0056
	Wet AMD	158.99	34.21	154.75	138.87–181.25	
	Dry AMD	167.16	43.84	158.12	132.62–188.94	

M—mean; SD—standard deviation; Me—median; Q—quartiles; AMD—age-related macular degeneration.

**Table 3:** Post hoc multiple comparisons of the choroidal thickness in analyzed groups controlled for age and gender (*p* values).

		Controls	Wet AMD	Dry AMD
Foveal (Central)	Controls	X	*p* < 0.0001	*p* < 0.0001
	Wet AMD	*p* < 0.0001	X	*p* = 0.8292
	Dry AMD	*p* < 0.0001	*p* = 0.8292	X
Perifoveal	Controls	X	*p* < 0.0001	*p* < 0.0001
	Wet AMD	*p* < 0.0001	X	*p* = 0.2021
	Dry AMD	*p* < 0.0001	*p* = 0.2021	X
Peripheral	Controls	X	*p* < 0.0001	*p* = 0.0021
	Wet AMD	*p* < 0.0001	X	*p* = 0.2867
	Dry AMD	*p* = 0.0021	*p* = 0.2867	X

**Table 4:** Comparison of choroidal thickness in wet AMD subgroups versus controls.

Analyzed Parameters	Study Group	Statistical Parameter				*p* Value
		*M*	*SD*	*Me*	*Q*_1_–*Q*_3_	
BCVA (logMAR)	Control Group	0.00	0.00	0.00	0.00–0.00	=0.7817
	Wet AMD + IVI	0.51	0.31	0.50	0.30–0.70	
	Wet AMD, IVI naïve	0.47	0.19	0.40	0.40–0.60	
Foveal (Central) Choroidal Thickness (µm)	Control Group	273.64	84.02	273.50	198.50–338.50	=0.0196
	Wet AMD + IVI	237.33	84.75	223.00	178.00–278.00	
	Wet AMD, IVI naïve	249.77	78.38	264.00	195.00–308.00	
Perifoveal Choroidal Thickness (µm)	Control Group	235.97	69.30	237.50	177.12–284.06	=0.0001
	Wet AMD + IVI	195.34	58.31	186.25	151.81–225.56	
	Wet AMD, IVI naïve	204.09	51.40	205.81	169.44–235.62	
Peripheral Choroidal Thickness (µm)	Control Group	177.51	45.96	166.62	143.31–203.37	=0.0057
	Wet AMD + IVI	157.68	35.92	151.25	132.62–179.25	
	Wet AMD, IVI naïve	166.97	33.27	158.37	153.50–178.37	

M—mean; SD—standard deviation; Me—median; Q—quartiles; AMD—age-related macular degeneration; IVI—intravitreal injections; BCVA—best-corrected visual acuity.

**Table 5:** Post hoc pair comparisons of choroidal thickness between wet AMD sub-cohorts and control group controlled for age and gender (*p* values).

		Controls	Wet AMD + IVI	Wet AMD, IVI Naïve
Foveal (Central)	Controls	X	*p* = 0.0083	*p* = 0.4456
	Wet AMD + IVI	*p* = 0.0083	X	*p* = 0.9245
	Wet AMD, IVI naïve	*p* = 0.4456	*p* = 0.9245	X
Perifoveal	Controls	X	*p* < 0.0001	*p* = 0.1309
	Wet AMD + IVI	*p* < 0.0001	X	*p* = 0.9336
	Wet AMD, IVI naïve	*p* = 0.1309	*p* = 0.9336	X
Peripheral	Controls	X	*p* = 0.0028	*p* = 0.4982
	Wet AMD + IVI	*p* = 0.0028	X	*p* = 0.8298
	Wet AMD, IVI naïve	*p* = 0.4982	*p* = 0.8298	X

AMD—age-related macular degeneration; IVI—intravitreal injections.

**Table 6:** Correlation coefficients with corresponding *p* values for selected traits in study participants’ eyes, controlled for age and gender.

Study Group	Wet AMD								Dry AMD			
Patient Characteristics	BCVA (logMAR)		Age (year)		IVI Count		Treatment Duration (months)		BCVA (logMAR)		Age (year)	
Measured field	r	*p*	r	*p*	r	*p*	r	*p*	r	*p*	r	*p*
Retinal Thickness												
Central	0.15	0.0856	0.01	0.8802	0.04	0.7109	–0.12	0.2556	–0.30	0.0018	–0.43	<0.0001
Perifoveal	–0.06	0.4856	–0.21	0.0204	–0.03	0.8060	–0.20	0.0426	–0.001	0.9927	–0.41	<0.0001
Peripheral	–0.10	0.2549	–0.25	0.0068	0.13	0.2426	–0.06	0.5918	–0.08	0.4114	–0.32	0.0008
Choroidal thickness												
Central	–0.16	0.0917	–0.24	0.0072	0.30	0.0044	0.13	0.1867	–0.11	0.2763	–0.41	<0.0001
Perifoveal	–0.11	0.2112	–0.23	0.0117	0.19	0.0836	0.06	0.5657	–0.07	0.4726	–0.46	<0.0001
Peripheral	–0.11	0.2443	–0.23	0.0100	0.14	0.1898	0.02	0.8808	–0.05	0.5826	–0.46	<0.0001
BCVA (logMAR)			0.24	0.0091	0.17	0.1193	0.21	0.0421			0.32	0.0010

BCVA—best-corrected visual acuity; IVI—intravitreal injections; r—rank coefficient.

## Data Availability

The original contributions presented in the study are included in the article; further inquiries can be directed to the corresponding author.

## References

[1] Xie R., Qiu B., Chhablani J., Zhang X. (2021). Evaluation of Choroidal Thickness Using Optical Coherent Tomography: A Review. Front. Med..

[2] Keane P.A., Liakopoulos S., Chang K.T., Wang M., Dustin L., Walsh A.C., Sadda S.R. (2008). Relationship between optical coherence tomography retinal parameters and visual acuity in neovascular age-related macular degeneration. Ophthalmology.

[3] Brandl C., Brücklmayer C., Günther F., Zimmermann M.E., Küchenhoff H., Helbig H., Weber B.H., Heid I.M., Stark K.J. (2019). Retinal Layer Thicknesses in Early Age-Related Macular Degeneration: Results From the German AugUR Study. Investig. Ophthalmol. Vis. Sci..

[4] Sodhi S.K., Golding J., Trimboli C., Choudhry N. (2021). Feasibility of peripheral OCT imaging using a novel integrated SLO ultra-widefield imaging swept-source OCT device. Int. Ophthalmol..

[5] Gawęcki M., Kiciński K. (2024). Advantages of the Utilization of Wide-Field OCT and Wide-Field OCT Angiography in Clinical Practice. Diagnostics.

[6] Tan C.S., Heussen F., Sadda S.R. (2013). Peripheral autofluorescence and clinical findings in neovascular and non-neovascular age-related macular degeneration. Ophthalmology.

[7] Lengyel I., Csutak A., Florea D., Leung I., Bird A.C., Jonasson F., Peto T. (2015). A Population-Based Ultra-Widefield Digital Image Grading Study for Age-Related Macular Degeneration-Like Lesions at the Peripheral Retina. Ophthalmology.

[8] Domalpally A., Clemons T.E., Danis R.P., Sadda S.R., Cukras C.A., Toth C.A., Friberg T.R., Chew E.Y., Writing Committee for the OPTOS PEripheral RetinA (OPERA) Study (Ancillary Study of Age-Related Eye Disease Study 2) (2017). Peripheral Retinal Changes Associated with Age-Related Macular Degeneration in the Age-Related Eye Disease Study 2: Age-Related Eye Disease Study 2 Report Number 12 by the Age-Related Eye Disease Study 2 Optos PEripheral RetinA (OPERA) Study Research Group. Ophthalmology.

[9] Cheung C.M.G., Lai T.Y.Y., Teo K., Ruamviboonsuk P., Chen S.J., Kim J.E., Gomi F., Koh A.H., Kokame G., Jordan-Yu J.M. (2021). Polypoidal Choroidal Vasculopathy: Consensus Nomenclature and Non-Indocyanine Green Angiograph Diagnostic Criteria from the Asia-Pacific Ocular Imaging Society PCV Workgroup. Ophthalmology.

[10] Salehi M.A., Nowroozi A., Gouravani M., Mohammadi S., Arevalo J.F. (2022). Associations of refractive errors and retinal changes measured by optical coherence tomography: A systematic review and meta-analysis. Surv. Ophthalmol..

[11] Jonas J.B., Forster T.M., Steinmetz P., Schlichtenbrede F.C., Harder B.C. (2014). Choroidal thickness in age-related macular degeneration. Retina.

[12] Manjunath V., Goren J., Fujimoto J.G., Duker J.S. (2011). Analysis of choroidal thickness in age-related macular degeneration using spectral-domain optical coherence tomography. Am. J. Ophthalmol..

[13] Ting D.S.W., Yanagi Y., Agrawal R., Teo H.Y., Seen S., Yeo I.Y.S., Mathur R., Chan C.M., Lee S.Y., Wong E.Y.M. (2017). Choroidal Remodeling in Age-related Macular Degeneration and Polypoidal Choroidal Vasculopathy: A 12-month Prospective Study. Sci. Rep..

[14] Mazaraki K., Fassnacht-Riederle H., Blum R., Becker M., Michels S. (2015). Change in choroidal thickness after intravitreal aflibercept in pretreated and treatment-naive eyes for neovascular age-related macular degeneration. Br. J. Ophthalmol..

[15] Ting D.S.W., Ng W.Y., Ng S.R., Tan S.P., San Yeo I.Y., Mathur R., Chan C.M., Tan A.C.S., Tan G.S.W., Wong T.Y. (2016). Choroidal Thickness Changes in Age-Related Macular Degeneration and Polypoidal Choroidal Vasculopathy: A 12-Month Prospective Study. Am. J. Ophthalmol..

[16] Jarmołkowska I., Figurska M., Rekas M. (2019). Choroidal Thickness Changes in Patients with Wet Age-related Macular Degeneration over One Year of Aflibercept Treatment. Klin. Oczna.

[17] Minnella A.M., Centini C., Gambini G., Savastano M.C., Pagliei V., Falsini B., Rizzo S., Ciasca G., Maceroni M. (2022). Choroidal Thickness Changes After Intravitreal Aflibercept Injections in Treatment-Naïve Neovascular AMD. Adv. Ther..

[18] Kumar J.B., Wai K.M., Ehlers J.P., Singh R.P., Rachitskaya A.V. (2019). Subfoveal choroidal thickness as a prognostic factor in exudative age-related macular degeneration. Br. J. Ophthalmol..

[19] McDonnell E.C., Heussen F.M., Ruiz-Garcia H., Ouyang Y., Narala R., Walsh A.C., Sadda S.R. (2014). Effect of anti-VEGF treatment on choroidal thickness over time in patients with neovascular age-related macular degeneration. Eur. J. Ophthalmol..

[20] Lee J.Y., Lee D.H., Lee J.Y., Yoon Y.H. (2013). Correlation Between Subfoveal Choroidal Thickness and the Severity or Progression of Nonexudative Age-Related Macular Degeneration. Investig. Ophthalmol. Vis. Sci..

[21] Sigler E.J., Randolph J.C. (2013). Comparison of Macular Choroidal Thickness Among Patients Older Than Age 65 With Early Atrophic Age-Related Macular Degeneration and Normals. Investig. Ophthalmol. Vis. Sci..

[22] Lindner M., Bezatis A., Czauderna J., Becker E., Brinkmann C.K., Schmitz-Valckenberg S., Fimmers R., Holz F.G., Fleckenstein M. (2015). Choroidal Thickness in Geographic Atrophy Secondary to Age-Related Macular Degeneration. Investig. Ophthalmol. Vis. Sci..

[23] Kiciński K., Gawęcki M. (2024). Choroidal and Retinal Thicknesses in Healthy Eyes Measured with Ultra-Wide-Field Optical Coherence Tomography. Diagnostics.

[24] Barresi C., Chhablani J., Dolz-Marco R., Gallego-Pinazo R., Berni A., Bandello F., Borrelli E. (2024). Retinal neurodegeneration in age-related macular degeneration. Eur. J. Ophthalmol..

[25] Pappuru R.R., Ouyang Y., Nittala M.G., Hemmati H.D., Keane P.A., Walsh A.C., Sadda S.R. (2011). Relationship between outer retinal thickness substructures and visual acuity in eyes with dry age-related macular degeneration. Investig. Ophthalmol. Vis. Sci..

[26] Acharya S., Kharel Sitaula R., Karki P., Mishra S.K., Dahal H.N., Poudel A. (2022). Does outer retinal layer thickness correlate with the central visual field indices in early dry age-related macular degeneration?. Taiwan J. Ophthalmol..

[27] Beer A.L., Plank T., Greenlee M.W. (2020). Aging and central vision loss: Relationship between the cortical macro-structure and micro-structure. Neuroimage.

[28] Ying G.S., Kim B.J., Maguire M.G., Huang J., Daniel E., Jaffe G.J., Grunwald J.E., Blinder K.J., Flaxel C.J., Rahhal F. (2014). Sustained visual acuity loss in the comparison of age-related macular degeneration treatments trials. JAMA Ophthalmol..

[29] Bhandari S., Konstantinou E., Agron E., Clemons T.E., Chew E.Y. (2022). Visual acuity results at ten years in participants with age-related macular degeneration enrolled in the Age-Related Eye Disease Study 2. Investig. Ophthalmol. Vis. Sci..

[30] Evans J.R., Fletcher A.E., Wormald R.P. (2004). Age-related macular degeneration causing visual impairment in people 75 years or older in Britain: An add-on study to the Medical Research Council Trial of Assessment and Management of Older People in the Community. Ophthalmology.

[31] Fukuda Y., Notomi S., Shiose S., Kano K., Hashimoto S., Fujiwara K., Akiyama M., Ishikawa K., Hisatomi T., Sonoda K.-H. (2023). Differences in Central and Peripheral Choroidal Thickness Among the Subtypes of Age-Related Macular Degeneration in an Asian Population. J. Clin. Med..

